# Nitric Oxide Synthase Inhibitors as Antidepressants

**DOI:** 10.3390/ph3010273

**Published:** 2010-01-20

**Authors:** Gregers Wegener, Vallo Volke

**Affiliations:** 1 Centre for Psychiatric Research, University of Aarhus, Skovagervej 2, DK-8240 Risskov, Denmark; 2 Department of Physiology, University of Tartu, Ravila 19, EE-70111 Tartu, Estonia; E-Mail: vallov@ut.ee (V.V.)

**Keywords:** nitric oxide, antidepressants, psychiatry, depression, anxiety

## Abstract

Affective and anxiety disorders are widely distributed disorders with severe social and economic effects. Evidence is emphatic that effective treatment helps to restore function and quality of life. Due to the action of most modern antidepressant drugs, serotonergic mechanisms have traditionally been suggested to play major roles in the pathophysiology of mood and stress-related disorders. However, a few clinical and several pre-clinical studies, strongly suggest involvement of the nitric oxide (NO) signaling pathway in these disorders. Moreover, several of the conventional neurotransmitters, including serotonin, glutamate and GABA, are intimately regulated by NO, and distinct classes of antidepressants have been found to modulate the hippocampal NO level *in vivo*. The NO system is therefore a potential target for antidepressant and anxiolytic drug action in acute therapy as well as in prophylaxis. This paper reviews the effect of drugs modulating NO synthesis in anxiety and depression.

## 1. Introduction

Recent data from Denmark and Europe [1,2], indicate that brain disorders account for 12% of all direct costs in the Danish health system and 9% of the total drug consumption was used for treatment of brain diseases. Expenses for brain diseases constituted 3% of the gross national product, and the total expenses for all investigated brain diseases were 37.3 billion DKK. Among brain disorders, affective disorders were among the most costly diseases, and anxiety disorders among the most prevalent.

The pathogenesis of mood disorders remains elusive, but it is evident that multiple factors, genetic and environmental, play a crucial role for adult psychopathology and neurobiology [3]. With regard to therapy, a significant proportion of affective disorder patients are partial or non responders, and there has been no major breakthrough in finding novel effective drug targets since the introduction of the current marketed antidepressant drugs in the 1950s to the 1980s, which all are based on monoaminergic pharmacological effects. Consequently, there exists a pressing need to develop novel treatment strategies and ultimately understand the etiology and pathophysiology of affective disorders.

Nitric Oxide, originally termed Endothelial-Derived Relaxing Factor (EDRF) before it was discovered that NO and EDRF were the same substance, serves important roles in the cardiovascular system and macrophages [4,5]. In addition, NO has been shown also to have an important role in the nervous system [6,7], where NO serves as a messenger molecule in a number of physiological processes, including processes being linked to the major psychiatric diseases [8,9,10,11]. The present paper will review general aspects of the NO system, as well as focus on inhibitors of NO production as putative therapeutic agents towards anxiety and affective disorders.

## 2. General aspects of Nitric Oxide

NO is a small molecule (MW 30 Da), which in vitro is a colorless gas and a product from the breakdown of N_2_. NO is degraded into nitrites and nitrates, and depending on the environmental conditions, the half life ranges from minutes to years [12]. The combination of one atom of N and one atom of O, results in the presence of an unpaired electron. However, NO is less reactive than many other free radicals, and does not react with itself. Nevertheless, the compound is known to be an important mediator of cytotoxicity in the immune system [13]. 

In biological systems the half-life of NO is estimated to be about 30 s or less [12]. The molecule is uncharged and is therefore freely diffusible across cell membranes and other structures. NO is produced and released by many different cells in multicellular organisms and can thus act as a tool for intercellular communication [14,15,16,17,18,19]. 

### 2.1. The Nitric Oxide Synthase enzymes

The enzyme responsible for the synthesis of NO, nitric oxide synthase (NOS), appears, in different isoforms which are constitutive or inducible. The activity of the constitutive NOS depends on Ca^2+^ and calmodulin, whereas the inducible NOS are independent from both Ca^2+^ and calmodulin. A distinction of the isoforms is also made based on the tissue where the NOS was identified the first time and primarily located. Of the constitutive isoforms, NOS in endothelial cells is mainly located in the cell membrane, and is termed eNOS. NOS in neuronal cells is located throughout the cell and termed nNOS. The inducible isoform, NOS in the immune system is located in macrophages is termed iNOS and consists of soluble and membrane bound NOS [19,20]. However, exceptions from this rule exist. nNOS has been found in a variety of non-neuronal cells and eNOS have been demonstrated in some neurons [21,22]. The present NOS classification thus consists of three classes, which does not specify the cells in which they may occur or whether they are induced, but refers to the tissue where the NOS was identified the first time:

- is the NOS first identified in neurons and which is dependent of elevated Ca^2+^.
- is the NOS which is independent of elevated Ca^2+^.
- is the NOS first identified in endothelial cells and which is dependent of elevated Ca^2+^.

### 2.2. Synthesis of NO

**Scheme 1:**
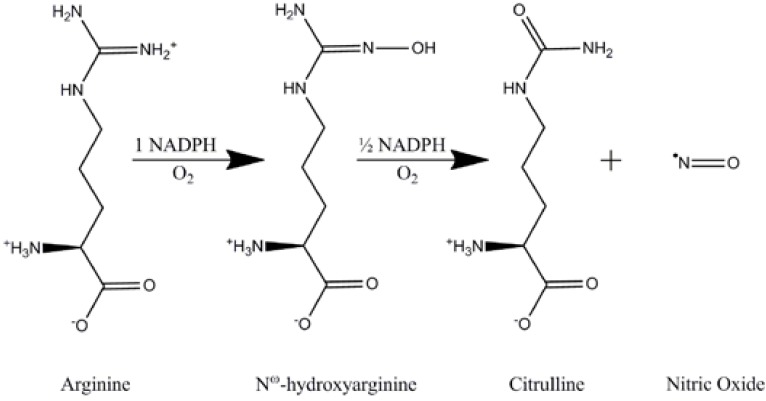
Synthesis of Nitric Oxide.

NO is synthesized in the brain by NOS from the amino-acid L-arginine. In brief, L-arginine is converted to N^ω^-hydroxy-L-arginine, which is further converted to NO and citrulline by NOS (Scheme 1). The process is rather complex and further discussion lies beyond the scope of this text. Briefly, the process involves five electrons, three co-substrates and five prostethic groups [19,23,24].

### 2.3. Localization of NOS in the CNS

The NOS enzymes are widely distributed within the mammalian brain [25,26]. The neuronal isoform accounts for the majority of the NOS activity in the brain [27], and NOS positive neurons are located in the hippocampal layers CA1-CA3, the medial amygdaloid nucleus, the olfactory bulb, the layers II-VI in the cerebral cortex, the granular and deep molecular layers of the cerebellum and, with special interest regarding the serotonin system, in the dorsal and medial raphe nuclei [25]. Measurements of NOS activity in different brain regions have shown the highest activity in the cerebellum, the midbrain, the hypothalamus, the cortex, the striatum and the hippocampus [28,29]. Interestingly, NO has been shown to co-localize with several other known transmitters within the same neuron, e.g. serotonin (5-HT) in the medial and dorsal raphe nuclei [30], Norepinephrine (NE) in the solitarian tract nucleus [31], γ-aminobutyric acid (GABA) in the cerebral cortex [32] and Neuropeptide Y (NPY) and somatostatin in the striatum [33].

It is important to emphasize that certain neurons also contain the eNOS besides the nNOS [34]. The consequences of this finding remain to be determined, but it is likely that neuronal eNOS and nNOS serve different roles in the CNS [35]. Under normal physiological conditions, iNOS in the brain should have no role, in that the activity of iNOS is largely undetectable. However, under pathological conditions, such as trauma, ischemia or infection, iNOS may become important [36]. 

### 2.4. Regulation of NOS activity

Regulation of the NOS enzyme expression has to be clarified in detail. Most of the studies performed have focused on the iNOS isoform. This isoform is not present in the cells under normal circumstances, but can be expressed following activation by different cytokines/endotoxines [37,38]. Less is known about the expression of nNOS and eNOS, but it has become evident that expression of nNOS in the brain and spinal-cord during the embryonic and post-natal period can change markedly, which is in line with evidence indicating that NO is implicated in synaptic plasticity in the adult and in regulating neurite outgrowth, as exemplified by the finding that NO donors enhance neurotrophin-induced neurite outgrowth through a cGMP-dependent mechanism and [39,40,41]. 

The co factors and especially the NOS-Ca^2+^-calmodulin interaction is a primary regulator for NO production. Following an action potential, increases in the intracellular Ca^2+^ environment (around 500 nM [42]), triggers Ca^2+^-calmodulin to bind to NOS, activating the NOS enzymatic activity. As, the intracellular Ca^2+^ level can rapidly change, the catalytic activity can be turned on and off within a short time. These regulatory properties form basis of the understanding of NO as a neurotransmitter. Interestingly, iNOS binds calmodulin very tightly, and continue to synthesize NO thoughout the life of the enzyme, irrespectively of the intracellular Ca^2+^ concentration [19]. In addition to the co-factor and Ca^2+^ level regulations, phosphorylation is used to regulate the activity, as exemplified by the finding that nNOS phosphorylation by protein kinase C inhibits NO production [43]. Finally, NO itself has been shown to regulate NOS activity [44,45,46]. The nature of this inhibition needs to be fully clarified, but can be hypothesized to involve nitrosylation [47].

### 2.5. Targets of NO 

NO has multiple targets in the brain, with the soluble form of the guanylate Cyclase (sGC) the most extensively characterized [38,48,49]. Activation of sGC subsequently increases the production of cGMP, and the level of cGMP in the cerebellum, striatum and hippocampus has been shown to depend largely on the NOS activity [50,51,52]. 

Some physiological effects of NO are, however, independent of sGC activation, and it has been demonstrated that NO, induced by NMDA receptor stimulation, activates the p21 (ras) pathway of signal transduction with a cascade involving extracellular signal-regulated kinases and phosphoinositide 3-kinase [53,54]. These pathways are known to be involved in transmission of signals to the cell nuclei and may therefore form a basis of a generation of long-lasting neuronal responses to NO. Other enzymes that constitute cellular targets for NO are cyclooxygenases, ribonucleotide reductase, some mitochondrial enzymes and NOS itself [55,56]. Finally, NO can nitrosylate proteins and damage the DNA [54,57,58,59].

## 3. NO and Psychiatric Disorders

Patients suffering from depression have been shown to have a reduced number of NOS containing neurons in the hypothalamus [60,61] and hippocampus [62]. In samples from suicide attempters, increased NO metabolites (NO_2_ and NO_3_) have been observed [63]. Moreover, a decreased platelet NOS activity and plasma NO metabolites in depressed patients [64,65] and a changed L-arginine metabolism in Bipolar Disorder have been reported [66]. In addition, human genetic association studies have repeatedly found association with NO signaling and psychiatric disorders [11,67].

## 4. NOS inhibitors: Evidence for Efficacy in Depression and Anxiety

Over the past two decades, a number of preclinical studies have demonstrated that inhibition of NOS produces anxiolytic and antidepressant-like behavioral effects in a variety of animal paradigms. These studies include systemic injections as well as targeted infusions into the brain. The studies are primarily acute studies, and there is a great need for examination of the chronic effects. Only a few very limited clinical studies are available, which are confounded by the nonselectivity of the drug used. However, as already mentioned there are several human studies indicating an important role of elevated NO in the pathogenesis of affective disorders and anxiety, suggesting that a positive role of inhibition may be possible. Below, the results from the different NOS inhibitors used are reviewed. See also Table 1.

### 4.1. NOS inhibiting amino acids.

The typical NOS inhibiting amino acids associate with the substrate binding site for L-arginine [68]. The inhibitor will compete with L-arginine, and usually extra arginine will reverse the NOS inhibition produced by the inhibitor.

The best investigated compounds in this family are L-NNA (L-NG-nitroarginine), its methyl ester L-NAME), L-NMMA (L-NG-monomethylarginine) and NG-propyl-L-arginine. L-NAME requires hydrolysis of the methyl ester by cellular esterases to become a fully functional inhibitor [68]. Acute antidepressant effects have been found in both rats and mice models. L-NNA and L-NAME have thus been reported to be effective in both the Forced Swim Test (FST) and Tail Suspension Test (TST) in mice [69,70], and in the FST in rats [71,72]. The effect of the drugs seems to display a U-shaped pharmacology, where both low and high doses have no effect [69,70,73]. Pretreatment with L-Arg has the ability to counteract the behavioral effects of the L-NAME and L-NNA [69,70,71,74], but has also been reported in some studies to have an antidepressant-like effect by itself [69]. 

**Table 1:** NOS inhibitors and studies in paradigms of depression and anxiety based on chemical class.

INHIBITORAMINO ACIDS	ENZYME/POTENCY	DRUG STRUCTURE	DEPRES-SION	ANXIETY	DRUG REF
L-NMMA or L-NANA (L-N^G^-Methyl-L-arginine)	nNOS=eNOS>>iNOS	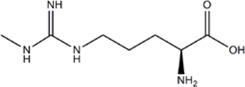	[70]	-	[68]
N-PLA (L-N^G-^Propyl-L-arginine)	nNOS>>eNOS>>iNOS	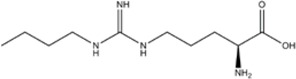	-	-	[75]
L-NNA (L-N^G^-Nitroarginine )	nNOS>eNOS>>>iNOS	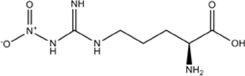	[69,70,72,76,77,78]	[79,80,81]	[68]
L-NAME (L-N^G^-Nitroarginine methyl ester)	nNOS>eNOS>iNOS	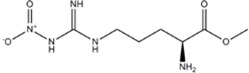	[70,71,82,83,84,85,86]	[81,87,88,89,90]	[68]
L-NAA NG-Amino-L-arginine	nNOS>eNOS>iNOS	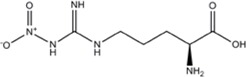	-	-	[91]
ADMA (N^G^,N^G^-Dimethyl-L-arginine)	-	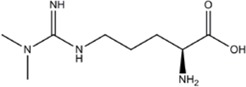	[92]	-	[93,94]
SDMA (NG,NG′-Dimethyl-L-arginine)	-	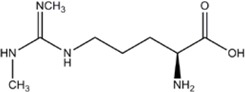			
L-NIL (L-N6-(1-Imino-ethyl)lysine)	iNOS>>nNOS	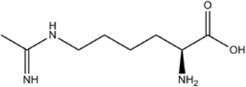	-	-	[95]
L-Thiocitrulline	nNOS>iNOS>eNOS	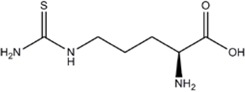	-	-	[96]
*S*-Methyl-L-Thiocitrulline	nNOS>eNOS>iNOS	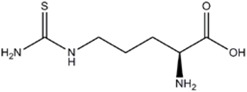	-	-	[97]
Agmatine (1-Amino-4-guanidinobutane)	Unspecific NOS inhibitor and ligand at imidazoline receptors	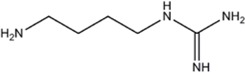	[98,99,100,101,102,103,104,105]	[102,106,107]	[108]
L-Canavanine	iNOS	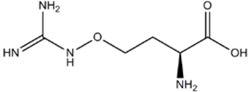	-	-	[109]
**AMIDINES**					
L-NIO Nδ-(Iminoethyl)-L-ornithine	nNOS>eNOS=iNOS	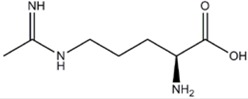	-	-	[110]
Ethyl-L-NIO	nNOS>iNOS>eNOS	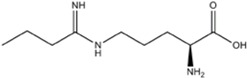	-	-	[111]
Vinyl-L-NIO	nNOS>>eNOS>iNOS	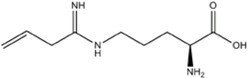	-	-	[111]
1400W (*N*-(3-(Aminomethyl)benzyl)acetamidine)	iNOS>>>nNOS>eNOS	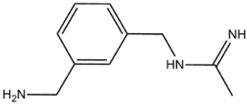	-	-	[112]
**INDAZOLE DERIVATES**					
7-NI (7-Nitroindazole)	nNOS=eNOS>>iNOS	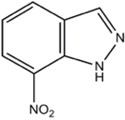	[72,89,113,114,115,116,117,118]	[89,119,120,121,122,123]	[124,125]
7-NI-Br (7-Bromonitroindazole)	nNOS>eNOS>iNOS	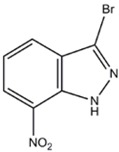			
**IMIDAZOLE DERIVATES**					
TRIM (1-[2-(Trifluoromethyl)phenyl-imidazole	iNOS=nNOS>eNOS	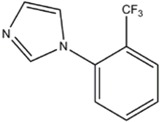	[115,126,127]	[128].	[129]
**2-IMINOPIPERIDINE DERIVATES**					
2-Imino-4-methylpiperidine	iNOS>nNOS>eNOS	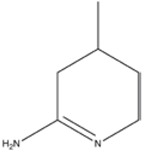	-	-	[130]
**HYDRAZINE DERIVATES**					
Aminoguanidine	iNOS>>nNOS	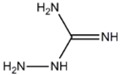	[98,131], [132]	[133]	[134]
**ISOTHIOUREAS**					
*S*-(2-Aminoethyl) isothiourea	iNOS=nNOS=eNOS	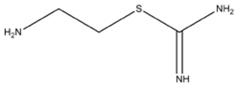	-	-	[135]
1,3-PBIT (S,S'-(1,3-Phenylene-bis(1,2-ethanediyl))bis-isothiourea)	iNOS>>nNOS>eNOS	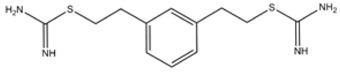	-	-	[135]
1,4-PBIT (S,S'-(1,4-Phenylene-bis(1,2-ethanediyl))bis-isothiourea)	iNOS>nNOS>>eNOS	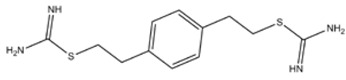	-	-	[135]
α-Guanidinoglutaric Acid	-	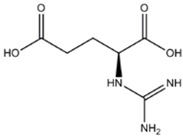	-	-	[136]
**OTHER/MIXED**					
Methylene blue	NNOS=eNOS=iNOS, sGC, MAO	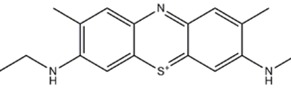	[118,137,138,139,140]	[137,141]	
ODQ ( [1H-[1,2,4]Oxadiazole[4,3-a]quinoxalin-1-one] -	Inhibits NO sensitive cGMP formation	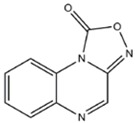	[116,142].	[121]	

The clinically important features in depression, cognition and memory, have been extensively examined, and a major role for NO in the formation of memory and as a mediator in synaptic plasticity has been suggested [143,144]. A majority of studies support a facilatory role of NO in learning processes, and nNOS has been proposed to be the principal source of this retrograde messenger during long-term potentiation (LTP) [22,145], a highly important process for memory formation [146,147,148]. However, some controversy about this finding exist, as LTP in hippocampus and cerebellum were reported to be normal in nNOS transgenic mice [34,149]. The involvement of NOS in memory has also been confirmed in studies with NOS inhibitors. For example it was shown that systemic administration L-NAME and L-NNA impairs acquisition but not retention of spatial learning in rats [76,83,84], and L-NA reduces hippocampal mediation of place learning in the rat [77,78]. Similarly, intrahippocampal administration of L-NAME impairs working memory on a runway task without affecting reference memory [85,86], and L-NAME has been shown to disrupt learning of an associative memory task, the conditioned eyeblink response in rabbits [83]. However, in a well-learned operant task–a delayed non-match-to-position, no effect of L-NAME was found [150], and similarly, it was also shown that L-NAME did not affect learning in a Morris Water Maze paradigm [151]. In agreement with these observations, central and systemic administration of the NO precursor, L-Arginine has been found to significantly prolong the latency time in the passive avoidance test without inhibition of locomotor and exploratory activity [152]. The interpretation of the overall neurobiological consequences of these findings remains to be established. The findings with NOS inhibitors do not initially seem correspond well with results published about other clinically relevant antidepressants, such as the SSRIs, where cognitive performance in patients have been shown to be unaffected [153] and independent from clinical recovery [154]. However, in a recent rodent study, it was reported that acute administration of imipramine and paroxetine to rats, impaired the discrimination of old from the recent objects [155]. Interestingly, following chronic administration, the imipramine-treated rats were unable to differentiate between the two objects, whereas paroxetine treated rats, as controls, spent more time exploring the old object [155]. Similarly it is, important to note that the studies with NOS inhibitors and cognitive testing predominantly have been carried out following one acute dose. The relevance for this paradigm related to a clinical context is, as it also is the case with the other depression and anxiety tests, questionable. Only limited information is available concerning chronic administration of NOS inhibitors. However, It has been shown that L-NAME in the drinking water over 14 days impairs working memory in rats [156]. On the other hand, it has also been demonstrated that only acute, but not chronic administration of L-NAME impairs LTP formation induced by a weak near-threshold tetanus [157]. Further studies must be carried out to conclude on the overall effects of NOS inhibition on cognition.

Some of the amino-acids require special attention, as they may be considered as endogenous inhibitors. These inhibitors include L-citrulline, agmatine, NG, NG-dimethyl-L-arginine (ADMA), and argininosuccinic acid. While L-citrulline is a very weak inhibitor, a derivate, L-thiocitrulline is much more powerful [96]. Agmatine is de-carboxylated arginine [158], and has gained significant attention as there is evidence of antidepressant effects in preclinical animal models of depression [99,101,102,103], as well as studies suggesting a key role of agmatine in humans [98,105,159,160]. It is here, however, noteworthy to mention that agmatine also has been conceptualized as an endogenous clonidine-displacing substance of imidazoline receptors [161,162], and to have affinity for several transmembrane receptors, such as α2-adrenergic [163], imidazoline I1 and glutamatergic NMDA receptors [108]. Therefore, the effects observed in the preclinical studies may be mediated via these pathways, and not linked to NOS. 

No solid preclinical data exist for the other endogenous inhibitors, although there are reports of their presence in animals [164]. Several human association studies have been published, especially regarding NG-monomethyl-L-arginine (SDMA) and ADMA [165,166,167]. Indeed, reports have shown an increased level of ADMA concentration in sepression, schizophrenia and Alzheimer’s disease [92,168,169]; however, it is not clear whether this association is clinically important. Taken together, despite the human studies predominantly are studies carried out on peripheral tissue samples (e.g. plasma or serum), a role for the NO system in psychiatric disease is supported. 

Within the field of anxiety, several interesting—but contradictory—findings have been observed, using different paradigms and drugs. For example, it has been suggested that NO has an anxiolytic-like action in the elevated plus maze (EPM) following administration of L-NNA [79,80], and also that inhibition with L-NNA caused an anxiolytic-like effect, and—in the same study—an anxiogenic-like effect with L-NAME [81]. In contrast, some other studies have reported potent anxiolytic-like effects of L-NAME in EPM [87,88,89]. Moreover, microinjections of L-NAME and L-NA into the periaqueductal grey were shown to produce anxiolytic-like-effects in EPM, an effect which was typically bell-shaped, and could be abolished by pre-treatment with L-arginine [90]. 

### 4.2. Indazoles and Imidazole derivates

Similar to the findings with the amino acids, antidepressant-like properties have also been demonstrated with the non-amino acid compounds. The primary benefit with the Indazoles and Imidazole derivates is a potential superiority in selectivity among the different isoforms of the NOS enzymes. This was first clear when 7-nitroindazole (7-NI) was discovered [170], as it did not have a profound effect on the blood pressure [124] as most of the amino acid inhibitors. Studies suggest that 7-NI not only interacts competitively at the substrate binding site in the NOS enzyme [170], but also competitive regarding the co-factor tetrahydrobiopterin (BH_4_) [171]. As 7-NI is also a potent inhibitor of bovine aortic endothelial eNOS in vitro, regardless of the lack of cardiovascular side-effects of this compound *in vivo* [170], other more selective isoform inhibitors have been screened. Such a compound is 1-(2-trifluoromethyphenyl)imidazole (TRIM), which is described as a potent and relatively selective inhibitor of nNOS both *in vivo* and *in vivo* [172,173]. The selectivity of this compound seems to be centered around the co-factor BH_4_, and the availability of BH_4_ in the tissues [129]. 

In the FST, 7-NI and TRIM has been found to be active [72,89,113,115,116] when administered acutely. There are no effects on locomotion following administration of the compounds. Interestingly, the effects of 7-NI have been shown to be centrally based, since intrahippocampal administration of 7-NI have been shown to cause a dose-dependent antidepressant-like effect in the FST, an effect which could be prevented following intra-hippocampal co-administration of L-arginine [114]. On the other depression related domains, 7-NI have been found to induce amnesia in a passive avoidance task in the chick [117], and impair learning and memory in different tasks such as the Morris water maze, radial maze, passive avoidance and elevated plus maze tests [123,174,175,177]. 7-NI have also been found to produce taste aversions, and enhance the lithium based taste aversion learning in a conditioned taste aversion paradigm, an effect that was counteracted with simultaneous administration of L-arginine [118]. 

Within the field of anxiety, there is more agreement on the findings with the indazoles and imidazole derivates, than with the amino acid inhibitors. It was thus shown that inhibition with 7-NI caused an anxiolytic-like effect in the EPM [89,120,122,123]. Also the selective nNOS inhibitor TRIM has been shown to possess anxiolytic-like effects in EPM [115], and has been found to modulate anxiety related behavior following the unpredictable chronic mild stress procedure in mice [128].

### 4.3. Hydrazine derivates and amidines

These compounds have been extensively studied in relation to cardiovascular [178,179,182] and endocrinological diseases [183,184,185,186]. The compounds are predominantly inhibitors of iNOS, with much less activity on the other isoforms. Aminoguanidine (AG) is a hydrazine derivate and the best characterized compound [187,188,189], which selectively decreases cGMP levels produced by iNOS [190]. Furthermore, AG has been observed to protect against neurodegeneration produced by chronic stress in rats [191], and to prevent the impairment of learning behavior and hippocampal long-term potentiation following transient cerebral ischemia in rats [192]. Interestingly, intracerebroventricular infusion of AG prevents the depression-like behavior following a chronic unpredictable stress paradigm [131]. Supporting these findings, a model of Post Traumatic Stress Disorder (PTSD) seems to involve exclusively the iNOS isoform, as only aminoguanidine, but not 7-NI, was effective in attenuating neurobiological readouts [132]. Together, these findings highlight the possible involvement of an inflammatory nature in depression and anxiety, which is not surprising due to the significant involvement of stress in the pathophysiology of the disorders. AG has also recently been demonstrated to display anxiolytic-like effects in EPM, open field test, light/dark test and social interaction test in stressed mice [133]. Whether these effects are present in the absence of stress remains to be established.

### 4.4. Other compounds/mixed

Within this group we find the only compounds proven to be effective in patients [139,140,193]. Methylene Blue (MB) oxidizes protein-bound heme and non-heme ferrous iron [194], inhibiting the stimulation of soluble guanylyl cyclase (sGC) by NO and nitrovasodilators [195]. MB was as early as 1899 described to have a calming—probably antipsychotic—effect in patients [196]. However, more recent work has focused on the beneficial effects of MB in manic-depressive disorder, where a response of 63% among 24 lithium refractory patients was found [138]. The studies were supplemented and expanded, confirming this action [139,140,193]. At the time of the study, the mechanistic hypotheses were based on changes in the vanadium ion [197,198,199,200]. Unfortunately, the studies cited above were not fully randomized, but luckily such trials are being carried out in these years [201]. It was in 1993 demonstrated that MB potently inhibited NOS both *in vitro* [202,203] and *in vivo* [204].

Several preclinical studies confirm a positive effect of MB in the FST and EPM [137], however with a U-shaped dose-response efficacy curve. Metylene blue have been demonstrated to produce taste aversions in a conditioned taste aversion paradigm, an effect comparable to the effects of 7-NI, which also could be cunteracted with simultaneous administration of L-arginine [118]. As indicated by the mode of action, MB is expected to be a very non-selective compound. Indeed, MB not only inhits NOS and sGC, but also several other heme containing enzymes, like mono-amine oxidase. In agreement with this, MB has been characterized as a potent inhibitor Monoamine Oxidase (MAO) [205,206,207] and various cytochromes. This effect is probably the explanation of case-reports suggesting a hyperserotonergic state following use of MB [206,208], and can be an explanation for the clinical efficacy.

Since MB also affects the NO downstream signaling pathway, including sGC, it is here worth to mention a few compounds mediating the, which affect sGC, but not NOS. Studies with selective (i.e. non-NOS) inhibitors of NO dependent cGMP formation with [1*H*-[1,2,4]oxadiazole[4,3-a]quinoxalin-1-one] (ODQ) have proven to produce antidepressant-like effects in the FST [116], as well as prevention of pro-depressant effect of L-arginine in the FST [142]. Similarly, ODQ have been shown to have anxiolytic-like properties, with an increase in the % time spent on the open arm in EPM following administration of the drug [121]. These findings are in agreement with other studies showing that an increase in cGMP, following inhibition of phosphodiesterase type V, using sildenafil, can produce anxiogenic-like responses in the EPM [209,210]. The mechanisms regarding cGMP may, however, not be easily understood, as also antidepressant actions of sildenafil have been shown following central muscarinic receptor blockade [211].

## 5. Interactions between NO and the Conventional Neurotransmitters

Several in vivo studies have demonstrated that NO may modulate the extracellular level of various neurotransmitters in the central nervous system, e.g. serotonin (5-HT), dopamine (DA), γ-aminobutyric acid (GABA), and glutamate [212,213,214,215,216,217,218]. In addition, NO can inactivate the rate limiting enzyme in the synthesis of 5-HT, tryptophan hydroxylase [219,220] and it has been suggested to stimulate synaptic vesicle release from hippocampal synaptosomes [221,222]. Furthermore, NO regulates 5-HT reuptake [223,224,225], inhibits uptake of [3H] DA by striatal synaptosomes [226,227] and transforms 5-HT into an inactive form [228] . More recently, it was demonstrated that a physical interaction between the serotonin transporter and neuronal nitric oxide synthase may underlie reciprocal modulation of their activity [229]. The connection between NO and 5-HT is substantiated by observations from neurology, where studies has shown that NO as well as 5-HT is involved in the pathophysiology of migraine [230,231,232,233]. 

Interestingly, it has also been reported that L-Arg antagonizes the effects of the classic tricyclic antidepressant, imipramine [70]. This observation has led to hypotheses regarding the potential contribution of serotonergic/noradrenergic mechanisms in the observed antidepressant-like effects of the NOS inhibitors. Subsequently, it has been demonstrated that low and ineffective doses of L-NAME were able to potentiate the behavioral effects of imipramine and fluoxetine but not reboxetine, a norepinephrin reuptake inhibitor, in the FST [72,234]. In addition, it was shown that a serotonergic mediation of the antidepressant-like effects of L-NA, 7-NI was present, since serotonergic depletion abolished the antidepressant-like effect of the inhibitors [72]. Not all inhibitors seem to display this profile, as it also was demonstrated that the effect of agmatine was independent of 5-HT depletion [99]. However, as already discussed, agmatine may have multiple effects on several receptorsystems. Finally, NO have also been implicated in the antidepressant role of several other substances, like tramadol [235], bupropion [236], and lithium [237]. Similarly, established antidepressants, like imipramine, paroxetine, citalopram and tianeptine have all been shown to inhibit hippocampal NOS activity in vivo when applied locally in the brain [238].

## 6. Conclusions

Although the studies cited in the current review utilize several different compounds, affecting the different isoforms of NOS differently, the physiological role of NOS inhibition remain relatively clear. Therefore, the conclusion of the current review is that despite significant challenges in developing compounds which may differentially inhibit the ‘right’ isoform at the right place, NOS inhibition continue to be an interesting novel approach in the future development of antidepressants. 

## References

[B1] Olesen J., Leonardi M. (2003). The burden of brain diseases in Europe. Eur. J. Neurol..

[B2] Olesen J., Sobscki P., Truelsen T., Sestoft D., Jonsson B. (2008). Cost of disorders of the brain in Denmark. Nord. J. Psychiatry.

[B3] Caspi A., Sugden K., Moffitt T.E., Taylor A., Craig I.W., Harrington H., McClay J., Mill J., Martin J., Braithwaite A., Poulton R. (2003). Influence of life stress on depression: moderation by a polymorphism in the 5-HTT gene. Science.

[B4] Palmer R.M., Ferrige A.G., Moncada S. (1987). Nitric oxide release accounts for the biological activity of endothelium-derived relaxing factor. Nature.

[B5] Hibbs Jr. J.B., Taintor R.R., Vavrin Z. (1987). Macrophage cytotoxicity: role for L-arginine deiminase and imino nitrogen oxidation to nitrite. Science.

[B6] Bredt D.S., Snyder S.H. (1989). Nitric oxide mediates glutamate-linked enhancement of cGMP levels in the cerebellum. Proc. Natl. Acad. Sci. USA.

[B7] Garthwaite J., Garthwaite G., Palmer R.M., Moncada S. (1989). NMDA receptor activation induces nitric oxide synthesis from arginine in rat brain slices. Eur. J. Pharmacol..

[B8] Knott A.B., Bossy-Wetzel E. (2009). Nitric oxide in health and disease of the nervous system. Antioxid. Redox Signal..

[B9] Oosthuizen F., Wegener G., Harvey B.H. (2005). Nitric oxide as inflammatory mediator in post-traumatic stress disorder (PTSD): evidence from an animal model. Neuropsychiat. Dis. Treatm..

[B10] Reif A., Herterich S., Strobel A., Ehlis A.C., Saur D., Jacob C.P., Wienker T., Topner T., Fritzen S., Walter U., Schmitt A., Fallgatter A.J., Lesch K.P. (2006). A neuronal nitric oxide synthase (NOS-I) haplotype associated with schizophrenia modifies prefrontal cortex function. Mol. Psychiatry.

[B11] Reif A., Jacob C.P., Rujescu D., Herterich S., Lang S., Gutknecht L., Baehne C.G., Strobel A., Freitag C.M., Giegling I., Romanos M., Hartmann A., Rosler M., Renner T.J., Fallgatter A.J., Retz W., Ehlis A.C., Lesch K.P. (2009). Influence of functional variant of neuronal nitric oxide synthase on impulsive behaviors in humans. Arch. Gen. Psychiatry.

[B12] Beckman J.S., Lancaster J. (1996). The Physiological and Pathological Chemistry of Nitric Oxide. In *Nitric Oxide: Principles and Actions*.

[B13] Stuehr D.J., Cho H.J., Kwon N.S., Weise M.F., Nathan C.F. (1991). Purification and characterization of the cytokine-induced macrophage nitric oxide synthase: an FAD- and FMN-containing flavoprotein. Proc. Natl. Acad. Sci. USA.

[B14] Bredt D.S., Snyder S.H. (1994). Nitric oxide: a physiologic messenger molecule. Annu. Rev. Biochem..

[B15] Garthwaite J., Charles S.L., Chess-Williams R. (1988). Endothelium-derived relaxing factor release on activation of NMDA receptors suggests role as intercellular messenger in the brain. Nature.

[B16] Kerwin Jr. J.F., Heller M. (1994). The arginine-nitric oxide pathway: a target for new drugs. Med. Res. Rev..

[B17] Marletta M.A. (1993). Nitric oxide synthase structure and mechanism. J. Biol. Chem..

[B18] Moncada S., Palmer R.M., Higgs E.A. (1989). Biosynthesis of nitric oxide from L-arginine. A pathway for the regulation of cell function and communication. Biochem. Pharmacol.

[B19] Nathan C. (1992). Nitric oxide as a secretory product of mammalian cells. FASEB J..

[B20] Forstermann U., Schmidt H.H., Pollock J.S., Sheng H., Mitchell J.A., Warner T.D., Nakane M., Murad F. (1991). Isoforms of nitric oxide synthase. Characterization and purification from different cell types. Biochem. Pharmacol..

[B21] Forstermann U., Gath I., Schwarz P., Closs E.I., Kleinert H. (1995). Isoforms of nitric oxide synthase. Properties, cellular distribution and expressional control. Biochem.Pharmacol..

[B22] O'Dell T.J., Hawkins R.D., Kandel E.R., Arancio O. (1991). Tests of the roles of two diffusible substances in long-term potentiation: evidence for nitric oxide as a possible early retrograde messenger. Proc. Natl. Acad. Sci. USA.

[B23] Dawson T.M., Snyder S.H. (1994). Gases as biological messengers: nitric oxide and carbon monoxide in the brain. J. Neurosci..

[B24] Knowles R.G., Moncada S. (1994). Nitric oxide synthases in mammals. Biochem. J..

[B25] Blottner D., Grozdanovic Z., Gossrau R. (1995). Histochemistry of nitric oxide synthase in the nervous system. Histochem.J..

[B26] de Vente J., Hopkins D.A., Markerink-Van I.M., Emson P.C., Schmidt H.H., Steinbusch H.W. (1998). Distribution of nitric oxide synthase and nitric oxide-receptive, cyclic GMP-producing structures in the rat brain. Neuroscience.

[B27] Hara H., Waeber C., Huang P.L., Fujii M., Fishman M.C., Moskowitz M.A. (1996). Brain distribution of nitric oxide synthase in neuronal or endothelial nitric oxide synthase mutant mice using [3H]L-NG-nitro-arginine autoradiography. Neuroscience.

[B28] Barjavel M.J., Bhargava H.N. (1995). Nitric oxide synthase activity in brain regions and spinal cord of mice and rats: kinetic analysis. Pharmacology.

[B29] Salter M., Duffy C., Garthwaite J., Strijbos P.J. (1995). Substantial regional and hemispheric differences in brain nitric oxide synthase (NOS) inhibition following intracerebroventricular administration of N omega-nitro-L-arginine (L-NA) and its methyl ester (L-NAME). Neuropharmacology.

[B30] Johnson M.D., Ma P.M. (1993). Localization of NADPH diaphorase activity in monoaminergic neurons of the rat brain. J. Comp. Neurol..

[B31] Simonian S.X., Herbison A.E. (1996). Localization of neuronal nitric oxide synthase-immunoreactivity within sub-populations of noradrenergic A1 and A2 neurons in the rat. Brain Res..

[B32] Valtschanoff J.G., Weinberg R.J., Kharazia V.N., Schmidt H.H., Nakane M., Rustioni A. (1993). Neurons in rat cerebral cortex that synthesize nitric oxide: NADPH diaphorase histochemistry, NOS immunocytochemistry, and colocalization with GABA. Neurosci. Lett..

[B33] Kowall N.W., Ferrante R.J., Beal M.F., Richardson Jr. E.P., Sofroniew M.V., Cuello A.C., Martin J.B. (1987). Neuropeptide Y, somatostatin, and reduced nicotinamide adenine dinucleotide phosphate diaphorase in the human striatum: a combined immunocytochemical and enzyme histochemical study. Neuroscience.

[B34] O'Dell T.J., Huang P.L., Dawson T.M., Dinerman J.L., Snyder S.H., Kandel E.R., Fishman M.C. (1994). Endothelial NOS and the blockade of LTP by NOS inhibitors in mice lacking neuronal NOS. Science.

[B35] Kano T., Shimizu-Sasamata M., Huang P.L., Moskowitz M.A., Lo E.H. (1998). Effects of nitric oxide synthase gene knockout on neurotransmitter release in vivo. Neuroscience.

[B36] Yoshida T., Waeber C., Huang Z., Moskowitz M.A. (1995). Induction of nitric oxide synthase activity in rodent brain following middle cerebral artery occlusion. Neurosci. Lett..

[B37] Nathan C., Xie Q.W. (1994). Regulation of biosynthesis of nitric oxide. J. Biol. Chem..

[B38] Schmidt H.H., Lohmann S.M., Walter U. (1993). The nitric oxide and cGMP signal transduction system: regulation and mechanism of action. Biochim. Biophys. Acta.

[B39] Hindley S., Juurlink B.H., Gysbers J.W., Middlemiss P.J., Herman M.A., Rathbone M.P. (1997). Nitric oxide donors enhance neurotrophin-induced neurite outgrowth through a cGMP-dependent mechanism. J. Neurosci. Res..

[B40] Contestabile A. (2000). Roles of NMDA receptor activity and nitric oxide production in brain development. Brain Res. Rev..

[B41] Hess D.T., Patterson S.I., Smith D.S., Skene J.H. (1993). Neuronal growth cone collapse and inhibition of protein fatty acylation by nitric oxide. Nature.

[B42] Schmidt H.H., Pollock J.S., Nakane M., Forstermann U., Murad F. (1992). Ca2+/calmodulin-regulated nitric oxide synthases. Cell Calcium.

[B43] Lowenstein C.J., Snyder S.H. (1992). Nitric oxide, a novel biologic messenger. Cell.

[B44] Assreuy J., Cunha F.Q., Liew F.Y., Moncada S. (1993). Feedback inhibition of nitric oxide synthase activity by nitric oxide. Br. J. Pharmacol..

[B45] Buga G.M., Griscavage J.M., Rogers N.E., Ignarro L.J. (1993). Negative feedback regulation of endothelial cell function by nitric oxide. Circ.Res..

[B46] Rengasamy A., Johns R.A. (1993). Regulation of nitric oxide synthase by nitric oxide. Mol. Pharmacol..

[B47] Gaston B.M., Carver J., Doctor A., Palmer L.A. (2003). S-nitrosylation signaling in cell biology. Mol. Interv..

[B48] Denninger J.W., Marletta M.A. (1999). Guanylate cyclase and the .NO/cGMP signaling pathway. Biochim. Biophys. Acta.

[B49] Miki N., Kawabe Y., Kuriyama K. (1977). Activation of cerebral guanylate cyclase by nitric oxide. Biochem. Biophys. Res. Commun..

[B50] Laitinen J.T., Laitinen K.S., Tuomisto L., Airaksinen M.M. (1994). Differential regulation of cyclic GMP levels in the frontal cortex and the cerebellum of anesthetized rats by nitric oxide: an in vivo microdialysis study. Brain Res..

[B51] Luo D., Vincent S.R. (1994). NMDA-dependent nitric oxide release in the hippocampus in vivo: interactions with noradrenaline. Neuropharmacology.

[B52] Vallebuona F., Raiteri M. (1994). Extracellular cGMP in the hippocampus of freely moving rats as an index of nitric oxide (NO) synthase activity. J. Neurosci..

[B53] Yun H.Y., Gonzalez-Zulueta M., Dawson V.L., Dawson T.M. (1998). Nitric oxide mediates N-methyl-D-aspartate receptor-induced activation of p21ras. Proc. Natl. Acad. Sci. USA.

[B54] Dawson T.M., Sasaki M., Gonzalez-Zulueta M., Dawson V.L. (1998). Regulation of neuronal nitric oxide synthase and identification of novel nitric oxide signaling pathways. *Prog*. Brain Res..

[B55] Dawson T.M., Dawson V.L. (1995). ADP-ribosylation as a mechanism for the action of nitric oxide in the nervous system. New Horiz..

[B56] Garthwaite J., Boulton C.L. (1995). Nitric oxide signaling in the central nervous system. Annu. Rev. Physiol.

[B57] Stamler J.S. (1994). Redox signaling: nitrosylation and related target interactions of nitric oxide. Cell.

[B58] Stamler J.S. (1995). S-nitrosothiols and the bioregulatory actions of nitrogen oxides through reactions with thiol groups. Curr. Top. Microbiol. Immunol..

[B59] Stamler J.S., Lamas S., Fang F.C. (2001). Nitrosylation. the prototypic redox-based signaling mechanism. Cell.

[B60] Bernstein H.G., Heinemann A., Krell D., Dobrowolny H., Bielau H., Keilhoff G., Bogerts B. (2005). Hypothalamic nitric oxide synthase in affective disorder: focus on the suprachiasmatic nucleus. Cell. Mol. Biol. (Noisy-le-grand).

[B61] Bernstein H.G., Stanarius A., Baumann B., Henning H., Krell D., Danos P., Falkai P., Bogerts B. (1998). Nitric oxide synthase-containing neurons in the human hypothalamus: reduced number of immunoreactive cells in the paraventricular nucleus of depressive patients and schizophrenics. Neuroscience.

[B62] Oliveira R.M., Guimaraes F.S., Deakin J.F. (2008). Expression of neuronal nitric oxide synthase in the hippocampal formation in affective disorders. Braz. J. Med. Biol. Res..

[B63] Lee B.H., Lee S.W., Yoon D., Lee H.J., Yang J.C., Shim S.H., Kim D.H., Ryu S.H., Han C., Kim Y.K. (2006). Increased plasma nitric oxide metabolites in suicide attempters. Neuropsychobiology.

[B64] Chrapko W.E., Jurasz P., Radomski M.W., Lara N., Archer S.L., Le Melledo J.M. (2004). Decreased platelet nitric oxide synthase activity and plasma nitric oxide metabolites in major depressive disorder. Biol. Psychiatry.

[B65] Chrapko W., Jurasz P., Radomski M.W., Archer S.L., Newman S.C., Baker G., Lara N., Le Melledo J.M. (2006). Alteration of decreased plasma NO metabolites and platelet NO synthase activity by paroxetine in depressed patients. Neuropsychopharmacol..

[B66] Yanik M., Vural H., Tutkun H., Zoroglu S.S., Savas H.A., Herken H., Kocyigit A., Keles H., Akyol O. (2004). The role of the arginine-nitric oxide pathway in the pathogenesis of bipolar affective disorder. Eur.Arch.Psychiatry Clin.Neurosci..

[B67] Reif A., Strobel A., Jacob C.P., Herterich S., Freitag C.M., Topner T., Mossner R., Fritzen S., Schmitt A., Lesch K.P. (2006). A NOS-III haplotype that includes functional polymorphisms is associated with bipolar disorder. Int. J. Neuropsychopharmacol..

[B68] Griffith O.W., Kilbourn R.G. (1996). Nitric oxide synthase inhibitors: amino acids. Methods Enzymol..

[B69] da Silva G.D., Matteussi A.S., dos Santos A.R., Calixto J.B., Rodrigues A.L. (2000). Evidence for dual effects of nitric oxide in the forced swimming test and in the tail suspension test in mice. Neuroreport.

[B70] Harkin A.J., Bruce K.H., Craft B., Paul I.A. (1999). Nitric oxide synthase inhibitors have antidepressant-like properties in mice. 1. Acute treatments are active in the forced swim test. Eur. J. Pharmacol..

[B71] Jefferys D., Funder J. (1996). Nitric oxide modulates retention of immobility in the forced swimming test in rats. Eur. J. Pharmacol..

[B72] Harkin A., Connor T.J., Walsh M., St John N., Kelly J.P. (2003). Serotonergic mediation of the antidepressant-like effects of nitric oxide synthase inhibitors. Neuropharmacol..

[B73] Karolewicz B., Paul I.A., Antkiewicz-Michaluk L. (2001). Effect of NOS inhibitor on forced swim test and neurotransmitters turnover in the mouse brain. Pol. J. Pharmacol..

[B74] Inan S.Y., Yalcin I., Aksu F. (2004). Dual effects of nitric oxide in the mouse forced swimming test: possible contribution of nitric oxide-mediated serotonin release and potassium channel modulation. Pharmacol. Biochem. Behav..

[B75] Zhang H.Q., Fast W., Marletta M.A., Martasek P., Silverman R.B. (1997). Potent and selective inhibition of neuronal nitric oxide synthase by N omega-propyl-L-arginine. J. Med. Chem..

[B76] Bohme G.A., Bon C., Lemaire M., Reibaud M., Piot O., Stutzmann J.M., Doble A., Blanchard J.C. (1993). Altered synaptic plasticity and memory formation in nitric oxide synthase inhibitor-treated rats. Proc. Natl. Acad. Sci. USA.

[B77] Mogensen J., Wortwein G., Gustafson B., Ermens P. (1995). L-nitroarginine reduces hippocampal mediation of place learning in the rat. Neurobiol. Learn. Mem..

[B78] Mogensen J., Wortwein G., Hasman A., Nielsen P., Wang Q. (1995). Functional and neurochemical profile of place learning after L-nitro-arginine in the rat. Neurobiol. Learn. Mem..

[B79] De Oliveira C.L., Del Bel E.A., Guimaraes F.S. (1997). Effects of L-NOARG on plus-maze performance in rats. Pharmacol. Biochem. Behav..

[B80] Czech D.A., Jacobson E.B., LeSueur-Reed K.T., Kazel M.R. (2003). Putative anxiety-linked effects of the nitric oxide synthase inhibitor L-NAME in three murine exploratory behavior models. Pharmacol. Biochem. Behav..

[B81] Pokk P., Vali M. (2002). The effects of the nitric oxide synthase inhibitors on the behaviour of small-platform-stressed mice in the plus-maze test. Prog. Neuropsychopharmacol. Biol. Psychiatry.

[B82] Sevgi S., Ozek M., Eroglu L. (2006). L-NAME prevents anxiety-like and depression-like behavior in rats exposed to restraint stress. Methods Find. Exp. Clin. Pharmacol..

[B83] Chapman P.F., Atkins C.M., Allen M.T., Haley J.E., Steinmetz J.E. (1992). Inhibition of nitric oxide synthesis impairs two different forms of learning. Neuroreport.

[B84] Estall L.B., Grant S.J., Cicala G.A. (1993). Inhibition of nitric oxide (NO) production selectively impairs learning and memory in the rat. Pharmacol. Biochem. Behav..

[B85] Ohno M., Yamamoto T., Watanabe S. (1993). Deficits in working memory following inhibition of hippocampal nitric oxide synthesis in the rat. Brain Res..

[B86] Ohno M., Yamamoto T., Watanabe S. (1994). Intrahippocampal administration of the NO synthase inhibitor L-NAME prevents working memory deficits in rats exposed to transient cerebral ischemia. Brain Res..

[B87] Volke V., Koks S., Vasar E., Bourin M., Bradwejn J., Mannisto P.T. (1995). Inhibition of nitric oxide synthase causes anxiolytic-like behaviour in an elevated plus-maze. Neuroreport.

[B88] Faria M.S., Muscara M.N., Moreno Junior H., Teixeira S.A., Dias H.B., De Oliveira B., Graeff F.G., De Nucci G. (1997). Acute inhibition of nitric oxide synthesis induces anxiolysis in the plus maze test. Eur. J. Pharmacol..

[B89] Spiacci Jr. A., Kanamaru F., Guimaraes F.S., Oliveira R.M. (2008). Nitric oxide-mediated anxiolytic-like and antidepressant-like effects in animal models of anxiety and depression. Pharmacol. Biochem. Behav..

[B90] Guimaraes F.S., de Aguiar J.C., Del Bel E.A., Ballejo G. (1994). Anxiolytic effect of nitric oxide synthase inhibitors microinjected into the dorsal central grey. Neuroreport.

[B91] Fukuto J.M., Wood K.S., Byrns R.E., Ignarro L.J. (1990). NG-amino-L-arginine: a new potent antagonist of L-arginine-mediated endothelium-dependent relaxation. Biochem. Biophys. Res. Commun..

[B92] Selley M.L. (2004). Increased (E)-4-hydroxy-2-nonenal and asymmetric dimethylarginine concentrations and decreased nitric oxide concentrations in the plasma of patients with major depression. J. Affect. Disord..

[B93] Matsuoka H., Itoh S., Kimoto M., Kohno K., Tamai O., Wada Y., Yasukawa H., Iwami G., Okuda S., Imaizumi T. (1997). Asymmetrical dimethylarginine, an endogenous nitric oxide synthase inhibitor, in experimental hypertension. Hypertension.

[B94] Matsuguma K., Ueda S., Yamagishi S., Matsumoto Y., Kaneyuki U., Shibata R., Fujimura T., Matsuoka H., Kimoto M., Kato S., Imaizumi T., Okuda S. (2006). Molecular mechanism for elevation of asymmetric dimethylarginine and its role for hypertension in chronic kidney disease. J. Am. Soc. Nephrol..

[B95] Moore W.M., Webber R.K., Jerome G.M., Tjoeng F.S., Misko T.P., Currie M.G. (1994). L-N6-(1-iminoethyl)lysine: a selective inhibitor of inducible nitric oxide synthase. J. Med. Chem..

[B96] Frey C., Narayanan K., McMillan K., Spack L., Gross S.S., Masters B.S., Griffith O.W. (1994). L-thiocitrulline. A stereospecific, heme-binding inhibitor of nitric-oxide synthases. J. Biol. Chem..

[B97] Narayanan K., Spack L., McMillan K., Kilbourn R.G., Hayward M.A., Masters B.S., Griffith O.W. (1995). S-alkyl-L-thiocitrullines. Potent stereoselective inhibitors of nitric oxide synthase with strong pressor activity in vivo. J. Biol. Chem..

[B98] Taksande B.G., Kotagale N.R., Tripathi S.J., Ugale R.R., Chopde C.T. (2009). Antidepressant like effect of selective serotonin reuptake inhibitors involve modulation of imidazoline receptors by agmatine. Neuropharmacology.

[B99] Krass M., Wegener G., Vasar E., Volke V. (2008). Antidepressant-like effect of agmatine is not mediated by serotonin. Behav. Brain Res..

[B100] Zomkowski A.D., Santos A.R., Rodrigues A.L. (2006). Putrescine produces antidepressant-like effects in the forced swimming test and in the tail suspension test in mice. Prog. Neuropsychopharmacol. Biol. Psychiatry.

[B101] Li Y.F., Gong Z.H., Cao J.B., Wang H.L., Luo Z.P., Li J. (2003). Antidepressant-like effect of agmatine and its possible mechanism. Eur. J. Pharmacol..

[B102] Aricioglu F., Altunbas H. (2003). Is agmatine an endogenous anxiolytic/antidepressant agent?. Ann. N. Y. Acad. Sci..

[B103] Zomkowski A.D., Hammes L., Lin J., Calixto J.B., Santos A.R., Rodrigues A.L. (2002). Agmatine produces antidepressant-like effects in two models of depression in mice. Neuroreport.

[B104] Dias Elpo Zomkowski A., Oscar Rosa A., Lin J., Santos A.R., Calixto J.B., Lucia Severo Rodrigues A. (2004). Evidence for serotonin receptor subtypes involvement in agmatine antidepressant like-effect in the mouse forced swimming test. Brain Res..

[B105] Halaris A., Zhu H., Feng Y., Piletz J.E. (1999). Plasma agmatine and platelet imidazoline receptors in depression. Ann. N. Y. Acad. Sci..

[B106] Gong Z.H., Li Y.F., Zhao N., Yang H.J., Su R.B., Luo Z.P., Li J. (2006). Anxiolytic effect of agmatine in rats and mice. Eur. J. Pharmacol..

[B107] Lavinsky D., Arteni N.S., Netto C.A. (2003). Agmatine induces anxiolysis in the elevated plus maze task in adult rats. Behav. Brain Res..

[B108] Yang X.C., Reis D.J. (1999). Agmatine selectively blocks the N-methyl-D-aspartate subclass of glutamate receptor channels in rat hippocampal neurons. J. Pharmacol. Exp. Ther..

[B109] Levy B., Valtier M., de Chillou C., Bollaert P.E., Cane D., Mallie J.P. (1999). Beneficial effects of L-canavanine, a selective inhibitor of inducible nitric oxide synthase, on lactate metabolism and muscle high energy phosphates during endotoxic shock in rats. Shock.

[B110] Rees D.D., Palmer R.M., Schulz R., Hodson H.F., Moncada S. (1990). Characterization of three inhibitors of endothelial nitric oxide synthase in vitro and in vivo. Br. J. Pharmacol..

[B111] Babu B.R., Griffith O.W. (1998). N5-(1-Imino-3-butenyl)-L-ornithine. A neuronal isoform selective mechanism-based inactivator of nitric oxide synthase. J. Biol. Chem..

[B112] Garvey E.P., Oplinger J.A., Furfine E.S., Kiff R.J., Laszlo F., Whittle B.J., Knowles R.G. (1997). 1400W is a slow, tight binding, and highly selective inhibitor of inducible nitric-oxide synthase in vitro and in vivo. J. Biol. Chem..

[B113] Yildiz F., Erden B.F., Ulak G., Utkan T., Gacar N. (2000). Antidepressant-like effect of 7-nitroindazole in the forced swimming test in rats. Psychopharmacology (Berl)..

[B114] Joca S.R., Guimaraes F.S. (2006). Inhibition of neuronal nitric oxide synthase in the rat hippocampus induces antidepressant-like effects. Psychopharmacology (Berl)..

[B115] Volke V., Wegener G., Bourin M., Vasar E. (2003). Antidepressant- and anxiolytic-like effects of selective neuronal NOS inhibitor 1-(2-trifluoromethylphenyl)-imidazole in mice. Behav. Brain Res..

[B116] Heiberg I.L., Wegener G., Rosenberg R. (2002). Reduction of cGMP and nitric oxide has antidepressant-like effects in the forced swimming test in rats. Behav. Brain Res..

[B117] Holscher C. (1994). 7-Nitro indazole, a neuron-specific nitric oxide synthase inhibitor, produces amnesia in the chick. Learn. Mem..

[B118] Wegener G., Volke V., Bandpey Z., Rosenberg R. (2001). Nitric oxide modulates lithium-induced conditioned taste aversion. Behav. Brain Res..

[B119] Yildiz F., Ulak G., Erden B.F., Gacar N. (2000). Anxiolytic-like effects of 7-nitroindazole in the rat plus-maze test. Pharmacol. Biochem. Behav..

[B120] Volke V., Soosaar A., Koks S., Bourin M., Mannisto P.T., Vasar E. (1997). 7-Nitroindazole, a nitric oxide synthase inhibitor, has anxiolytic-like properties in exploratory models of anxiety. Psychopharmacology (Berl)..

[B121] Spolidorio P.C., Echeverry M.B., Iyomasa M., Guimaraes F.S., Del Bel E.A. (2007). Anxiolytic effects induced by inhibition of the nitric oxide-cGMP pathway in the rat dorsal hippocampus. Psychopharmacology (Berl)..

[B122] Dunn R.W., Reed T.A., Copeland P.D., Frye C.A. (1998). The nitric oxide synthase inhibitor 7-nitroindazole displays enhanced anxiolytic efficacy without tolerance in rats following subchronic administration. Neuropharmacology.

[B123] Yildiz Akar F., Ulak G., Tanyeri P., Erden F., Utkan T., Gacar N. (2007). 7-Nitroindazole, a neuronal nitric oxide synthase inhibitor, impairs passive-avoidance and elevated plus-maze memory performance in rats. Pharmacol. Biochem. Behav..

[B124] Moore P.K., Babbedge R.C., Wallace P., Gaffen Z.A., Hart S.L. (1993). 7-Nitro indazole, an inhibitor of nitric oxide synthase, exhibits anti-nociceptive activity in the mouse without increasing blood pressure. Br. J. Pharmacol..

[B125] Bland-Ward P.A., Moore P.K. (1995). 7-Nitro indazole derivatives are potent inhibitors of brain, endothelium and inducible isoforms of nitric oxide synthase. Life Sci..

[B126] Matsumura N., Kikuchi-Utsumi K., Nakaki T. (2008). Activities of 7-nitroindazole and 1-(2-(trifluoromethylphenyl)-imidazole independent of neuronal nitric-oxide synthase inhibition. J. Pharmacol. Exp. Ther..

[B127] Ulak G., Mutlu O., Akar F.Y., Komsuoglu F.I., Tanyeri P., Erden B.F. (2008). Neuronal NOS inhibitor 1-(2-trifluoromethylphenyl)-imidazole augment the effects of antidepressants acting via serotonergic system in the forced swimming test in rats. Pharmacol. Biochem. Behav..

[B128] Mutlu O., Ulak G., Laugeray A., Belzung C. (2009). Effects of neuronal and inducible NOS inhibitor 1-[2-(trifluoromethyl) phenyl] imidazole (TRIM) in unpredictable chronic mild stress procedure in mice. Pharmacol. Biochem. Behav..

[B129] Handy R.L., Moore P.K. (1997). Mechanism of the inhibition of neuronal nitric oxide synthase by 1-(2- trifluoromethylphenyl) imidazole (TRIM). Life Sci..

[B130] Webber R.K., Metz S., Moore W.M., Connor J.R., Currie M.G., Fok K.F., Hagen T.J., Hansen D.W., Jerome G.M., Manning P.T., Pitzele B.S., Toth M.V., Trivedi M., Zupec M.E., Tjoeng F.S. (1998). Substituted 2-iminopiperidines as inhibitors of human nitric oxide synthase isoforms. J. Med. Chem..

[B131] Wang D., An S.C., Zhang X. (2008). Prevention of chronic stress-induced depression-like behavior by inducible nitric oxide inhibitor. Neurosci. Lett..

[B132] Harvey B.H., Oosthuizen F., Brand L., Wegener G., Stein D.J. (2004). Stress-restress evokes sustained iNOS activity and altered GABA levels and NMDA receptors in rat hippocampus. Psychopharmacology.

[B133] Gilhotra N., Dhingra D. (2009). Involvement of NO-cGMP pathway in anti-anxiety effect of aminoguanidine in stressed mice. Prog. Neuropsychopharmacol. Biol. Psychiatry.

[B134] Joly G.A., Ayres M., Chelly F., Kilbourn R.G. (1994). Effects of NG-methyl-L-arginine, NG-nitro-L-arginine, and aminoguanidine on constitutive and inducible nitric oxide synthase in rat aorta. Biochem. Biophys. Res. Commun..

[B135] Garvey E.P., Oplinger J.A., Tanoury G.J., Sherman P.A., Fowler M., Marshall S., Harmon M.F., Paith J.E., Furfine E.S. (1994). Potent and selective inhibition of human nitric oxide synthases. Inhibition by non-amino acid isothioureas. J. Biol. Chem..

[B136] Yokoi I., Kabuto H., Habu H., Mori A. (1994). alpha-Guanidinoglutaric acid, an endogenous convulsant, as a novel nitric oxide synthase inhibitor. J. Neurochem..

[B137] Eroglu L., Caglayan B. (1997). Anxiolytic and antidepressant properties of methylene blue in animal models. Pharmacol. Res..

[B138] Narsapur S.L., Naylor G.J. (1983). Methylene blue. A possible treatment for manic depressive psychosis. J. Affect. Disord..

[B139] Naylor G.J., Martin B., Hopwood S.E., Watson Y. (1986). A two-year double-blind crossover trial of the prophylactic effect of methylene blue in manic-depressive psychosis. Biol. Psychiatry.

[B140] Naylor G.J., Smith A.H., Connelly P. (1987). A controlled trial of methylene blue in severe depressive illness. Biol. Psychiatry.

[B141] Savegnago L., Jesse C.R., Pinto L.G., Rocha J.B., Barancelli D.A., Nogueira C.W., Zeni G. (2008). Diphenyl diselenide exerts antidepressant-like and anxiolytic-like effects in mice: involvement of L-arginine-nitric oxide-soluble guanylate cyclase pathway in its antidepressant-like action. Pharmacol. Biochem. Behav..

[B142] Ergun Y., Ergun U.G. (2007). Prevention of pro-depressant effect of L-arginine in the forced swim test by NG-nitro-L-arginine and [1H-[1,2,4]Oxadiazole[4,3-a]quinoxalin-1-one]. Eur. J. Pharmacol..

[B143] Prast H., Philippu A. (2001). Nitric oxide as modulator of neuronal function. Prog. Neurobiol..

[B144] Papa M., Pellicano M.P., Sadile A.G. (1994). Nitric oxide and long-term habituation to novelty in the rat. Ann. N. Y. Acad. Sci..

[B145] Schuman E.M., Madison D.V. (1991). A requirement for the intercellular messenger nitric oxide in long-term potentiation. Science.

[B146] Bliss T.V.P., Collingridge G.L. (1993). A synaptic model of memory: Long-term potentiation in the hippocampus. Nature.

[B147] Madison D.V., Malenka R.C., Nicoll R.A. (1991). Mechanisms underlying long-term potentiation of synaptic transmission. Annu. Rev. Neurosci..

[B148] Malenka R.C. (1994). Synaptic plasticity in the hippocampus: LTP and LTD. Cell.

[B149] Linden D.J., Dawson T.M., Dawson V.L. (1995). An evaluation of the nitric oxide/cGMP/cGMP-dependent protein kinase cascade in the induction of cerebellar long-term depression in culture. J. Neurosci..

[B150] Wiley J.L., Willmore C.B. (2000). Effects of nitric oxide synthase inhibitors on timing and short-term memory in rats. Behav. Pharmacol..

[B151] Bannerman D.M., Chapman P.F., Kelly P.A., Butcher S.P., Morris R.G. (1994). Inhibition of nitric oxide synthase does not impair spatial learning. J. Neurosci..

[B152] Plech A., Klimkiewicz T., Maksym B. (2003). Effect of L-arginine on memory in rats. Pol. J. Pharmacol..

[B153] Siepmann M., Grossmann J., Muck-Weymann M., Kirch W. (2003). Effects of sertraline on autonomic and cognitive functions in healthy volunteers. Psychopharmacology (Berl)..

[B154] Herrera-Guzman I., Gudayol-Ferre E., Herrera-Guzman D., Guardia-Olmos J., Hinojosa-Calvo E., Herrera-Abarca J.E. (2009). Effects of selective serotonin reuptake and dual serotonergic-noradrenergic reuptake treatments on memory and mental processing speed in patients with major depressive disorder. J. Psychiatr. Res..

[B155] Naudon L., Hotte M., Jay T.M. (2007). Effects of acute and chronic antidepressant treatments on memory performance: a comparison between paroxetine and imipramine. Psychopharmacology (Berl)..

[B156] Cobb B.L., Ryan K.L., Frei M.R., Guel-Gomez V., Mickley G.A. (1995). Chronic administration of L-NAME in drinking water alters working memory in rats. Brain Res. Bull..

[B157] Bannerman D.M., Chapman P.F., Kelly P.A., Butcher S.P., Morris R.G. (1994). Inhibition of nitric oxide synthase does not prevent the induction of long-term potentiation in vivo. J. Neurosci..

[B158] Wiesinger H. (2001). Arginine metabolism and the synthesis of nitric oxide in the nervous system. Prog. Neurobiol..

[B159] Halaris A., Piletz J.E. (2001). Imidazoline receptors: possible involvement in the pathophysiology and treatment of depression. Hum. Psychopharmacol. Clin. Exp..

[B160] Halaris A., Piletz J.E. (2003). Relevance of imidazoline receptors and agmatine to psychiatry: a decade of progress. Ann. N. Y. Acad. Sci..

[B161] Regunathan S., Reis D.J. (1996). Imidazoline receptors and their endogenous ligands. Annu. Rev. Pharmacol. Toxicol..

[B162] Li G., Regunathan S., Barrow C.J., Eshraghi J., Cooper R., Reis D.J. (1994). Agmatine: an endogenous clonidine-displacing substance in the brain. Science.

[B163] Olmos G., DeGregorio-Rocasolano N., Paz Regalado M., Gasull T., Assumpcio Boronat M., Trullas R., Villarroel A., Lerma J., Garcia-Sevilla J.A. (1999). Protection by imidazol(ine) drugs and agmatine of glutamate-induced neurotoxicity in cultured cerebellar granule cells through blockade of NMDA receptor. Br. J. Pharmacol..

[B164] Ogawa T., Kimoto M., Watanabe H., Sasaoka K. (1987). Metabolism of NG,NG-and NG,N'G-dimethylarginine in rats. Arch. Biochem. Biophys..

[B165] Boger R.H., Diemert A., Schwedhelm E., Luneburg N., Maas R., Hecher K. (2009). The Role of Nitric Oxide Synthase Inhibition by Asymmetric Dimethylarginine in the Pathophysiology of Preeclampsia. Gynecol. Obstet. Invest..

[B166] Boger R.H., Maas R., Schulze F., Schwedhelm E. (2009). Asymmetric dimethylarginine (ADMA) as a prospective marker of cardiovascular disease and mortality-An update on patient populations with a wide range of cardiovascular risk. Pharmacol. Res..

[B167] Boger R.H., Sullivan L.M., Schwedhelm E., Wang T.J., Maas R., Benjamin E.J., Schulze F., Xanthakis V., Benndorf R.A., Vasan R.S. (2009). Plasma asymmetric dimethylarginine and incidence of cardiovascular disease and death in the community. Circulation.

[B168] Das I., Khan N.S., Puri B.K., Hirsch S.R. (1996). Elevated endogenous nitric oxide synthase inhibitor in schizophrenic plasma may reflect abnormalities in brain nitric oxide production. Neurosci. Lett..

[B169] Arlt S., Schulze F., Eichenlaub M., Maas R., Lehmbeck J.T., Schwedhelm E., Jahn H., Boger R.H. (2008). Asymmetrical dimethylarginine is increased in plasma and decreased in cerebrospinal fluid of patients with Alzheimer's disease. Dement. Geriatr. Cogn. Disord..

[B170] Babbedge R.C., Bland-Ward P.A., Hart S.L., Moore P.K. (1993). Inhibition of rat cerebellar nitric oxide synthase by 7-nitro indazole and related substituted indazoles. Br. J. Pharmacol..

[B171] Mayer B., Klatt P., Werner E.R., Schmidt K. (1994). Molecular mechanisms of inhibition of porcine brain nitric oxide synthase by the antinociceptive drug 7-nitro-indazole [published erratum appears in Neuropharmacology 1995 Feb;34(2):243]. Neuropharmacology.

[B172] Handy R.L., Wallace P., Gaffen Z.A., Whitehead K.J., Moore P.K. (1995). The antinociceptive effect of 1-(2-trifluoromethylphenyl) imidazole (TRIM), a potent inhibitor of neuronal nitric oxide synthase in vitro, in the mouse. Br. J. Pharmacol..

[B173] Handy R.L., Harb H.L., Wallace P., Gaffen Z., Whitehead K.J., Moore P.K. (1996). Inhibition of nitric oxide synthase by 1-(2-trifluoromethylphenyl) imidazole (TRIM) in vitro: antinociceptive and cardiovascular effects. Br. J. Pharmacol..

[B174] Holscher C., McGlinchey L., Anwyl R., Rowan M.J. (1996). 7-Nitro indazole, a selective neuronal nitric oxide synthase inhibitor in vivo, impairs spatial learning in the rat. Learn. Mem..

[B175] Mizuno M., Yamada K., Olariu A., Nawa H., Nabeshima T. (2000). Involvement of brain-derived neurotrophic factor in spatial memory formation and maintenance in a radial arm maze test in rats. J. Neurosci..

[B176] Zou L.B., Yamada K., Tanaka T., Kameyama T., Nabeshima T. (1998). Nitric oxide synthase inhibitors impair reference memory formation in a radial arm maze task in rats. Neuropharmacology.

[B177] Yildiz Akar F., Celikyurt I.K., Ulak G., Mutlu O. (2009). Effects of L-arginine on 7-nitroindazole-induced reference and working memory performance of rats. Pharmacology.

[B178] Yang B., Larson D.F., Watson R.R. (2004). Modulation of iNOS activity in age-related cardiac dysfunction. Life Sci..

[B179] Ishibashi Y., Shimada T., Murakami Y., Takahashi N., Sakane T., Sugamori T., Ohata S., Inoue S., Ohta Y., Nakamura K., Shimizu H., Katoh H., Hashimoto M. (2001). An inhibitor of inducible nitric oxide synthase decreases forearm blood flow in patients with congestive heart failure. J. Am. Coll. Cardiol..

[B180] Wang D., Yang X.P., Liu Y.H., Carretero O.A., LaPointe M.C. (1999). Reduction of myocardial infarct size by inhibition of inducible nitric oxide synthase. Am. J. Hypertens..

[B181] Takano H., Manchikalapudi S., Tang X.L., Qiu Y., Rizvi A., Jadoon A.K., Zhang Q., Bolli R. (1998). Nitric oxide synthase is the mediator of late preconditioning against myocardial infarction in conscious rabbits. Circulation.

[B182] Gardiner S.M., Kemp P.A., March J.E., Bennett T. (1996). Influence of aminoguanidine and the endothelin antagonist, SB 209670, on the regional haemodynamic effects of endotoxaemia in conscious rats. Br. J. Pharmacol..

[B183] Rydgren T., Sandler S. (2002). Efficacy of 1400 W, a novel inhibitor of inducible nitric oxide synthase, in preventing interleukin-1beta-induced suppression of pancreatic islet function in vitro and multiple low-dose streptozotocin-induced diabetes in vivo. Eur. J. Endocrinol..

[B184] Suarez-Pinzon W.L., Mabley J.G., Strynadka K., Power R.F., Szabo C., Rabinovitch A. (2001). An inhibitor of inducible nitric oxide synthase and scavenger of peroxynitrite prevents diabetes development in NOD mice. J. Autoimmun..

[B185] Shimabukuro M., Ohneda M., Lee Y., Unger R.H. (1997). Role of nitric oxide in obesity-induced beta cell disease. J. Clin. Invest..

[B186] Holstad M., Jansson L., Sandler S. (1996). Effects of aminoguanidine on rat pancreatic islets in culture and on the pancreatic islet blood flow of anaesthetized rats. Biochem. Pharmacol..

[B187] Corbett J.A., McDaniel M.L. (1996). The Use of Aminoguanidine, a Selective iNOS Inhibitor, to Evaluate the Role of Nitric Oxide in the Development of Autoimmune Diabetes. Methods.

[B188] Hasan K., Heesen B.J., Corbett J.A., McDaniel M.L., Chang K., Allison W., Wolffenbuttel B.H., Williamson J.R., Tilton R.G. (1993). Inhibition of nitric oxide formation by guanidines. Eur. J. Pharmacol..

[B189] Griffiths M.J., Messent M., MacAllister R.J., Evans T.W. (1993). Aminoguanidine selectively inhibits inducible nitric oxide synthase. Br. J. Pharmacol..

[B190] Griffiths M.J., Messent M., Curzen N.P., Evans T.W. (1995). Aminoguanidine selectively decreases cyclic GMP levels produced by inducible nitric oxide synthase. Am. J. Respir. Crit. Care Med..

[B191] Olivenza R., Moro M.A., Lizasoain I., Lorenzo P., Fernandez A.P., Rodrigo J., Bosca L., Leza J.C. (2000). Chronic stress induces the expression of inducible nitric oxide synthase in rat brain cortex. J. Neurochem..

[B192] Mori K., Togashi H., Ueno K.I., Matsumoto M., Yoshioka M. (2001). Aminoguanidine prevented the impairment of learning behavior and hippocampal long-term potentiation following transient cerebral ischemia. Behav. Brain Res..

[B193] Naylor G.J., Smith A.H., Connelly P. (1988). Methylene blue in mania [letter]. Biol. Psychiatry.

[B194] Salaris S.C., Babbs C.F., Voorhees W.D. (1991). Methylene blue as an inhibitor of superoxide generation by xanthine oxidase. A potential new drug for the attenuation of ischemia/reperfusion injury. Biochem. Pharmacol..

[B195] Murad F., Mittal C.K., Arnold W.P., Katsuki S., Kimura H. (1978). Guanylate cyclase: activation by azide, nitro compounds, nitric oxide, and hydroxyl radical and inhibition by hemoglobin and myoglobin. Adv. Cyclic Nucleotide Res..

[B196] Bodoni P. (1899). Le bleu de mÃ©thylÃ¨ne comme calmant chez le aliÃ©nÃ©s. La Semaine Médicale.

[B197] Naylor G.G., Smith A.H. (1982). Reduction of vanadate, a possible explanation of the effect of phenothiazines in manic-depressive psychosis. Lancet.

[B198] Naylor G.G., Smith A.H. (1982). Reduction of vanadate, a possible explanation of the effect of phenothiazines in manic-depressive psychosis [letter]. Lancet.

[B199] Naylor G.J. (1984). Vanadium and manic depressive psychosis. Nutr. Health.

[B200] Naylor G.J., Dick D.A., Johnston B.B., Hopwood S.E., Dick E.G., Smith A.H., Kay D. (1981). Possible explanation for therapeutic action of lithium, and a possible substitute (methylene-blue) [letter]. Lancet.

[B201] Alda M. NCT00214877: Methylene Blue for Cognitive Dysfunction in Bipolar Disorder. NCT00214877.

[B202] Mayer B., Brunner F., Schmidt K. (1993). Inhibition of nitric oxide synthesis by methylene blue. Biochem.Pharmacol..

[B203] Mayer B., Brunner F., Schmidt K. (1993). Novel actions of methylene blue. Eur.Heart J..

[B204] Volke V., Wegener G., Vasar E., Rosenberg R. (1999). Methylene blue inhibits hippocampal nitric oxide synthase activity in vivo. Brain Res..

[B205] Ehringer H., Hornykiewicz O., Lechner K. (1961). Die Wirkung von Methylenblau auf die Monoaminoxydase und den Katecholamin-und 5-Hydroxytryptaminstoffwechsel des Gehirnes. Naunyn. Schmiedebergs Arch. Exp. Pathol. Pharmakol..

[B206] Gillman P.K. (2008). Methylene blue is a potent monoamine oxidase inhibitor. Can. J. Anaesth..

[B207] Jakubovic A., Necina J. (1963). The effect of methylene blue on the monoamine oxidase activity of the liver and brain of rats after various routes of administration. Arzneimittelforschung.

[B208] Stanford S.C., Stanford B.J., Gillman P.K. (2009). Risk of severe serotonin toxicity following co-administration of methylene blue and serotonin reuptake inhibitors: an update on a case report of post-operative delirium. J. Psychopharmacol. (Oxf)..

[B209] Kurt M., Bilge S.S., Aksoz E., Kukula O., Celik S., Kesim Y. (2004). Effect of sildenafil on anxiety in the plus-maze test in mice. Pol. J. Pharmacol..

[B210] Volke V., Wegener G., Vasar E. (2003). Augmentation of the NO-CGMP cascade induces anxiogenic-like effect in mice. J. Physiol. Pharmacol..

[B211] Brink C.B., Clapton J.D., Eagar B.E., Harvey B.H. (2008). Appearance of antidepressant-like effect by sildenafil in rats after central muscarinic receptor blockade: evidence from behavioural and neuro-receptor studies. J. Neural Transm..

[B212] Kaehler S.T., Singewald N., Sinner C., Philippu A. (1999). Nitric oxide modulates the release of serotonin in the rat hypothalamus. Brain Res..

[B213] Lorrain D.S., Hull E.M. (1993). Nitric oxide increases dopamine and serotonin release in the medial preoptic area. Neuroreport.

[B214] Segovia G., Del Arco A., Mora F. (1997). Endogenous glutamate increases extracellular concentrations of dopamine, GABA, and taurine through NMDA and AMPA/kainate receptors in striatum of the freely moving rat: a microdialysis study. J. Neurochem..

[B215] Segovia G., Del Arco A., Mora F. (1999). Role of glutamate receptors and glutamate transporters in the regulation of the glutamate-glutamine cycle in the awake rat. Neurochem. Res..

[B216] Segovia G., Porras A., Mora F. (1994). Effects of a nitric oxide donor on glutamate and GABA release in striatum and hippocampus of the conscious rat. Neuroreport.

[B217] Strasser A., McCarron R.M., Ishii H., Stanimirovic D., Spatz M. (1994). L-arginine induces dopamine release from the striatum in vivo. Neuroreport.

[B218] Wegener G., Volke V., Rosenberg R. (2000). Endogenous nitric oxide decreases hippocampal levels of serotonin and dopamine in vivo. Br. J. Pharmacol..

[B219] Kuhn D.M., Arthur Jr. R. (1997). Molecular mechanism of the inactivation of tryptophan hydroxylase by nitric oxide: attack on critical sulfhydryls that spare the enzyme iron center. J. Neurosci..

[B220] Kuhn D.M., Arthur Jr. R.E. (1996). Inactivation of brain tryptophan hydroxylase by nitric oxide. J. Neurochem..

[B221] Meffert M.K., Calakos N.C., Scheller R.H., Schulman H. (1996). Nitric oxide modulates synaptic vesicle docking fusion reactions. Neuron.

[B222] Meffert M.K., Premack B.A., Schulman H. (1994). Nitric oxide stimulates Ca(2+)-independent synaptic vesicle release. Neuron.

[B223] Pogun S., Baumann M.H., Kuhar M.J. (1994). Nitric oxide inhibits [3H]dopamine uptake. Brain Res..

[B224] Pogun S., Dawson V., Kuhar M.J. (1994). Nitric oxide inhibits 3H-glutamate transport in synaptosomes. Synapse.

[B225] Pogun S., Kuhar M.J. (1994). Regulation of neurotransmitter reuptake by nitric oxide. Ann. N.Y. Acad. Sci..

[B226] Lonart G., Cassels K.L., Johnson K.M. (1993). Nitric oxide induces calcium-dependent [3H]dopamine release from striatal slices. J Neurosci. Res..

[B227] Lonart G., Johnson K.M. (1994). Inhibitory effects of nitric oxide on the uptake of [3H]dopamine and [3H]glutamate by striatal synaptosomes. J. Neurochem..

[B228] Fossier P., Blanchard B., Ducrocq C., Leprince C., Tauc L., Baux G. (1999). Nitric oxide transforms serotonin into an inactive form and this affects neuromodulation. Neuroscience.

[B229] Chanrion B., Mannoury la Cour C., Bertaso F., Lerner-Natoli M., Freissmuth M., Millan M.J., Bockaert J., Marin P. (2007). Physical interaction between the serotonin transporter and neuronal nitric oxide synthase underlies reciprocal modulation of their activity. Proc. Natl. Acad. Sci. USA.

[B230] Lassen L.H., Ashina M., Christiansen I., Ulrich V., Grover R., Donaldson J., Olesen J. (1998). Nitric oxide synthase inhibition: A new principle in the treatment of migraine attacks. Cephalgia.

[B231] Lassen L.H., Ashina M., Christiansen I., Ulrich V., Olesen J. (1997). Nitric oxide synthase inhibition in migraine [letter]. Lancet.

[B232] Thomsen L.L. (1997). Investigations into the role of nitric oxide and the large intracranial arteries in migraine headache. Cephalgia.

[B233] Thomsen L.L., Olesen J. (1998). Nitric oxide theory of migraine. Clin.Neurosci..

[B234] Harkin A., Connor T.J., Burns M.P., Kelly J.P. (2004). Nitric oxide synthase inhibitors augment the effects of serotonin re-uptake inhibitors in the forced swimming test. Eur. Neuropsychopharmacol..

[B235] Jesse C.R., Bortolatto C.F., Savegnago L., Rocha J.B., Nogueira C.W. (2008). Involvement of L-arginine-nitric oxide-cyclic guanosine monophosphate pathway in the antidepressant-like effect of tramadol in the rat forced swimming test. Prog. Neuropsychopharmacol. Biol. Psychiatry.

[B236] Dhir A., Kulkarni S.K. (2007). Involvement of nitric oxide (NO) signaling pathway in the antidepressant action of bupropion, a dopamine reuptake inhibitor. Eur. J. Pharmacol..

[B237] Ghasemi M., Sadeghipour H., Mosleh A., Sadeghipour H.R., Mani A.R., Dehpour A.R. (2008). Nitric oxide involvement in the antidepressant-like effects of acute lithium administration in the mouse forced swimming test. Eur. Neuropsychopharmacol..

[B238] Wegener G., Volke V., Harvey B.H., Rosenberg R. (2003). Local, but not systemic, administration of serotonergic antidepressants decreases hippocampal nitric oxide synthase activity. Brain Res..

